# Relaxation and Performance During Microsurgical Learning

**DOI:** 10.1177/22925503231184267

**Published:** 2023-07-18

**Authors:** Lara Cortellini, Antoine Nogueira, Thierry Christen, Justine Lattion, Sébastien Durand

**Affiliations:** 1 Department of Hand Surgery, Lausanne University Hospital and University of Lausanne, Lausanne, Switzerland; 2 Département des technologies industrielles, Haute Ecole d'Ingénierie et de Gestion du canton de Vaud ( 118538 HEIG-VD), Yverdon-les-Bains, Switzerland

**Keywords:** microsurgery, anastomosis, training, surgical competence, dexterity, relaxation, Microchirurgie, anastomose, relaxation, compétence chirurgicale, formation, dextérité

## Abstract

**Introduction:** Microsurgical learning is a difficult and stressful process, requiring self-control to achieve relaxation. The purpose of this study is to evaluate peripheral and central nervous system relaxation during microsurgical training. **Methods:** This cohort study included ten medical students with no previous experience in microsurgery. The somatic peripheral nervous system was evaluated by the force applied to a custom-designed microsurgical needle holder. The autonomic peripheral nervous system was assessed by a heart rate monitor. Central nervous system relaxation was evaluated by the State and Trait Anxiety Inventory scores. The quality of the anastomosis was graded by the Microsurgical Anastomosis Rating Scale (MARS10). These data were compared to a group of 5 senior microsurgeons who underwent a single assessment. **Results:** The time to complete the anastomosis and the force decreased significantly with training after only 2 weeks (*P* < .05). After 4 weeks of training, no statistical difference was observed between students and experts regarding force while the time of suture was still significantly different at 30 days (*P* = .001). The maximum heart rate decreased significantly at 2 weeks (*P* = .01). Anxiety scores decreased significantly between days 1 and 15 (*P* = .002 and *P* = .036). The MARS10 score demonstrates that the quality of the suture increases significantly during the first 15 days (*P* = .006). **Conclusion**: Peripheral and central nervous system relaxation as well as the quality of the microsurgical anastomosis increase significantly after only 15 days of learning. The force-sensing microsurgical needle holder offers a new tool for the evaluation of relaxation and can function as a learning aid.

## Résumé

**Introduction:** L’apprentissage de la microchirurgie est un processus difficile et stressant nécessitant un contrôle de soi permettant de se détendre. L’objectif de cette étude est d’évaluer la relaxation du système nerveux central et périphérique pendant la formation à la microchirurgie. **Méthodes:** cette cohorte d’étude a inclus dix étudiants en médecine sans expérience antérieure de la microchirurgie. Le système nerveux périphérique somatique a été évalué par la force appliquée sur un porte-aiguille microchirurgical conçu spécialement. Le système nerveux périphérique autonome a été évalué au moyen d’un moniteur de fréquence cardiaque. La relaxation du système nerveux central a été évaluée par des cotes de l’inventaire d’état et de trait anxieux (STAI: State and Trait Anxiety Inventory). La qualité des anastomoses a été cotée avec l’échelle MARS10. Ces données ont été comparées à celles d’un groupe de cinq chirurgiens expérimentés en microchirurgie et soumis à une évaluation unique. **Résultats:** Le temps nécessaire à la réalisation d’une anastomose complète et la force utilisée ont diminué significativement avec l’entraînement après seulement 2 semaines (*P* <.05). Après quatre semaines de formation, on n’observait plus de différence statistique entre les étudiants et les experts pour la force appliquée, tandis que le temps de suture était encore significativement différent à 30 jours (*P* = .001). La fréquence cardiaque maximum avait significativement diminué à 2 semaines (*P* = .01). Les scores d’anxiété ont diminué significativement entre les jours 1 et 15 (*P* = .002 et *P* = .036). Le score MARS10 démontre que la qualité des sutures augmente significativement au cours des 15 premiers jours (*P* = .006). **Conclusion:** La relaxation du système nerveux central et du système nerveux périphérique, ainsi que la qualité des anastomoses microchirurgicales augmentent significativement après seulement 15 jours d’apprentissage. Le porte-aiguille microchirurgical à capteur de force constitue un nouvel outil pour l’évaluation de la relaxation et peut servir d’aide à l’apprentissage.

## Introduction

In the past years, microsurgical training has been based on the “Halstedian” model which is a nonreproducible learning method and a subjective assessment model.^1^ Several studies have aimed to improve microsurgical teaching methods as well as develop various objective assessment tools for trainee surgeons. They are based on global rating scales and checklists,^2‐5^ hand motion analysis,^3,4,6‐9^ assessment of vessel patency,^8,10^ vessel physiological function, and virtual reality simulator.^11‐13^ In addition to assessing the quality of the microsurgical suture, objective instruments are critical to properly evaluate the handling of the instruments and to provide instructors with proper tools for microsurgical teaching.

Relaxation is the physical and mental change resulting from a decrease in muscle and nervous tension, a generalized diminution of neurophysiological excitation.^14^ Surgical performance can be enhanced by low levels of stress and increased with relaxation.^15,16^ Microsurgery learning, demanding high precision and accuracy, is difficult and stressful, requiring physical, emotional, and intellectual self-control to achieve relaxation.^17^

The aim of this study was to evaluate the peripheral and central nervous system relaxation during microsurgical training. In order to perform a multiparametric evaluation, we have developed a force-sensing microsurgical needle holder and used tools already available in our research platform or in the literature.^18^

We sought to demonstrate that relaxation increases as microsurgical training progress and that it is correlated to the quality of the microsurgical suture.

## Methods

### Subjects

Ten healthy volunteers (5 women and 5 men) with an average age of 25 years (range: 22–31), all right handed, were selected. They were all fifth-year medical students with no prior microsurgical experience. Exclusion criteria were individuals that suffered from pain or disability of the upper limbs or medical conditions modifying their ability to perform precise manual tasks, those with a history of hand surgery, participants taking medications that can affect motor skills and students with a hierarchical link to the investigators. Five expert microsurgeons were assessed as a control group.

### Microsurgical training protocol and tests

Approval was obtained from the local ethics committee (commission cantonale d’éthique de la recherche sur l’être humain).

Before the first training sessions, a macro-demonstration lesson was provided for end-to-end anastomosis according to Buncke.^19^ Microsurgical training included 2 weekly 1-hour sessions for 4 weeks and for students only. The expert microsurgeons result only used as a reference. An end-to-end anastomosis was performed on the brachial artery of a chicken wing under a Zeiss OPMI 111 microscope at 12.5× magnification using nonresorbable dafilon 10-0.^20^

**Figure 1.**
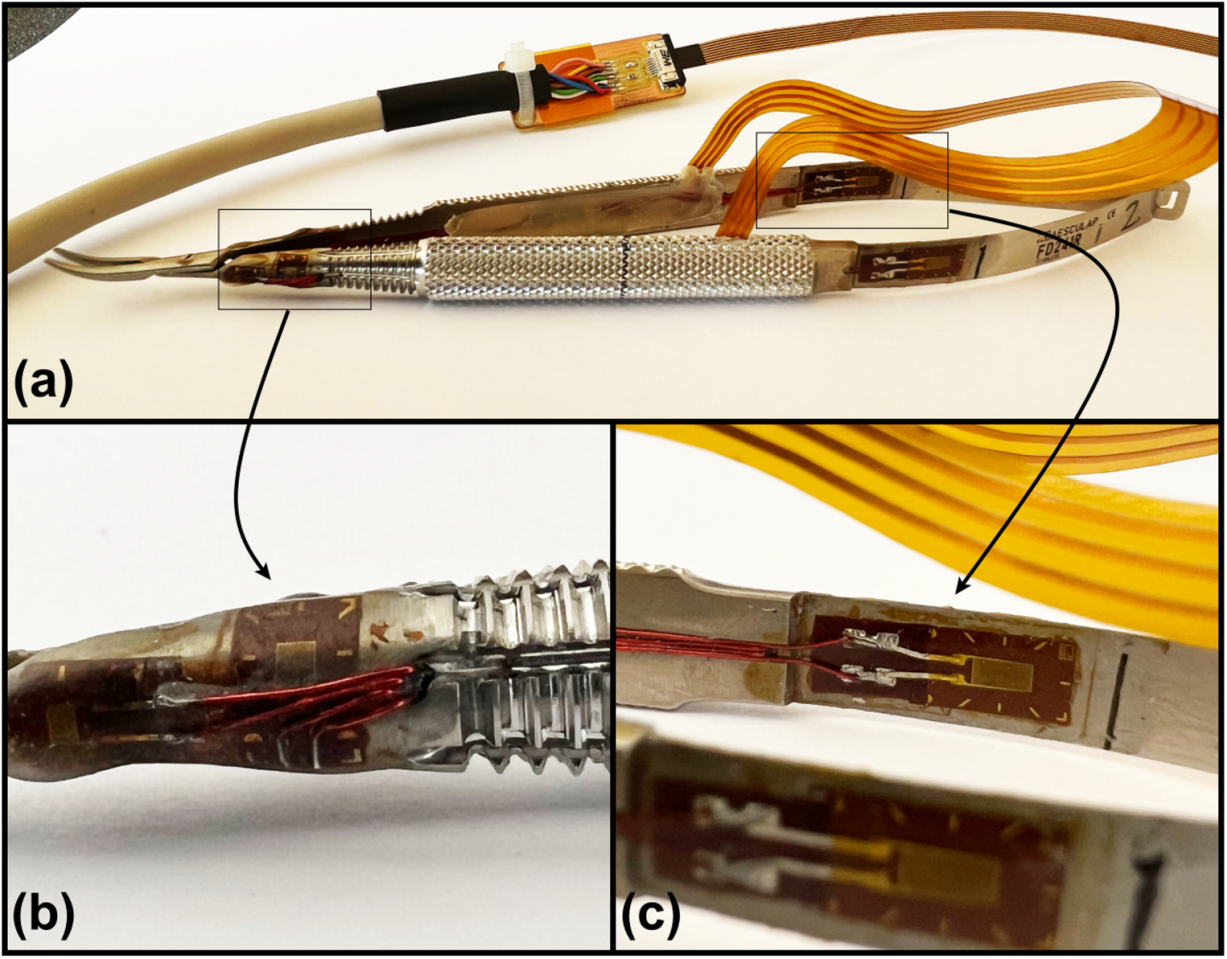
Force-sensing microsurgical needle holder (a), 8 strain gauges are glued and soldered to a flex printed circuit. Four 1-LY11-0.6/120 strain gauges (HBM, Zug, Switzerland) are glued between the screw and the grooves of each handle and placed on the outer side (b). Four 1-LY13-3/350 strain gauge (HBM, Zug, Switzerland) are glued at each side of each spring strips at the level of the maximum deformation (c).

Relaxation assessment occurred at 3 different time-points (days 1, 15, and 30) while performing a single microsuture, from grabing the needle to completion of the last knot. Somatic peripheral nervous system relaxation was assessed using a custom-made force-sensing microsurgical needle holder (Figure 1). Sensors included in a microsurgical needle holder FD 241 (Aesculap, Center Valley, PA, USA) allow measurement of the force applied on the spring strips, see Durand et al^18^ for full details on this device.

Central nervous system relaxation was evaluated by the State-Trait Anxiety Inventory (STAI) questionnaire. STAI consists of 2 questionnaires of twenty self-reported items each. The first questionnaire (STAI Form Y-1) measures how participants feel “at this moment” which allows evaluation of the nervousness and the anxiety during the session (for example “I feel calm, I feel nervous”). The second questionnaire (STAI Form Y-2) reflects the usual emotional state.^21^

An ECG tracing was also acquired using a simple 1 channel thoracic belt ECG system connected through a Bluetooth link to a mobile telephone with the Movesense Showcase App and then transferred to the laptop computer as an Excel file. The ECG used was the MAX-ECG Monitor from Maxim Integrated.

During the same training period, the quality of the microsurgical anastomosis was assessed using the Microsurgical Anastomosis Rating Scale (MARS10) score, which is a validated tool to assess microsurgical end-to-end arterial anastomoses on nonliving models.^22^ It consists of 5 items (anastomosis closure, suture spacing, bites size, knot tying, and cut end length), graded on a 3 points scale (0–2 points). Blinded evaluation of the MARS10 score was performed for each student by 2 experts in microsurgery.

### Statistics

Statistical analysis was performed using the Wilcoxon signed-rank test and the Spearman correlation test. A *p*-value < .05 was considered statistically significant.

## Results

**Figure 2.**
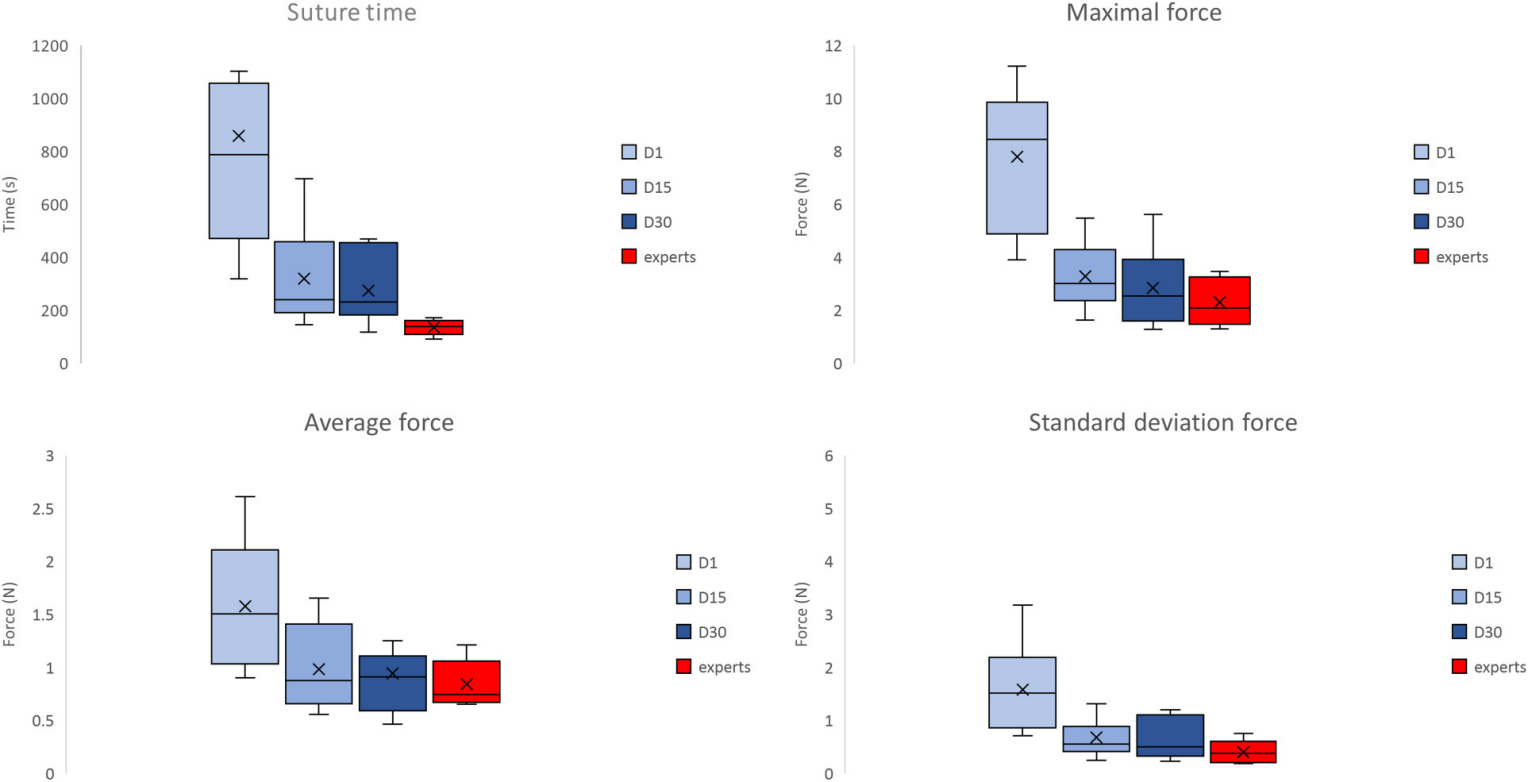
Suture time, maximal force, average force and standard deviation force for students at day 1, day 15 and day 30 in students and in experts. *p < .05.

The maximal force applied to the microsurgical needle holder decreases significantly (Figure 2) with training at day 15 (*p* = .002) and the difference is no longer significant between days 15 and 30 (*p* = .16). In comparison with experts, the difference is highly significant at day 1 (p = 0.0005) but this difference is no longer significant at day 15 (*p* = .25).

The average force (Figure 2) during suturing decreases significantly between days 1 and 15 (*p* = .006) but is no longer significant between days 15 and 30 (*p* = .77). In comparison with the experts, the difference is highly significant at day 1 (*p* = .008) but this difference is no longer significant at day 15 (*p* = .59).

The standard deviation of the force applied to the needle holder (Figure 2) during the suture decreases significantly between days 1 and 15 (*p* = .002) but no longer between days 15 and 30 (*p* = .37). In comparison with the experts, the difference is highly significant at day 1 (*p* = .001) but this difference is no longer significant at day 15 (*p* = .13).

**Figure 3.**
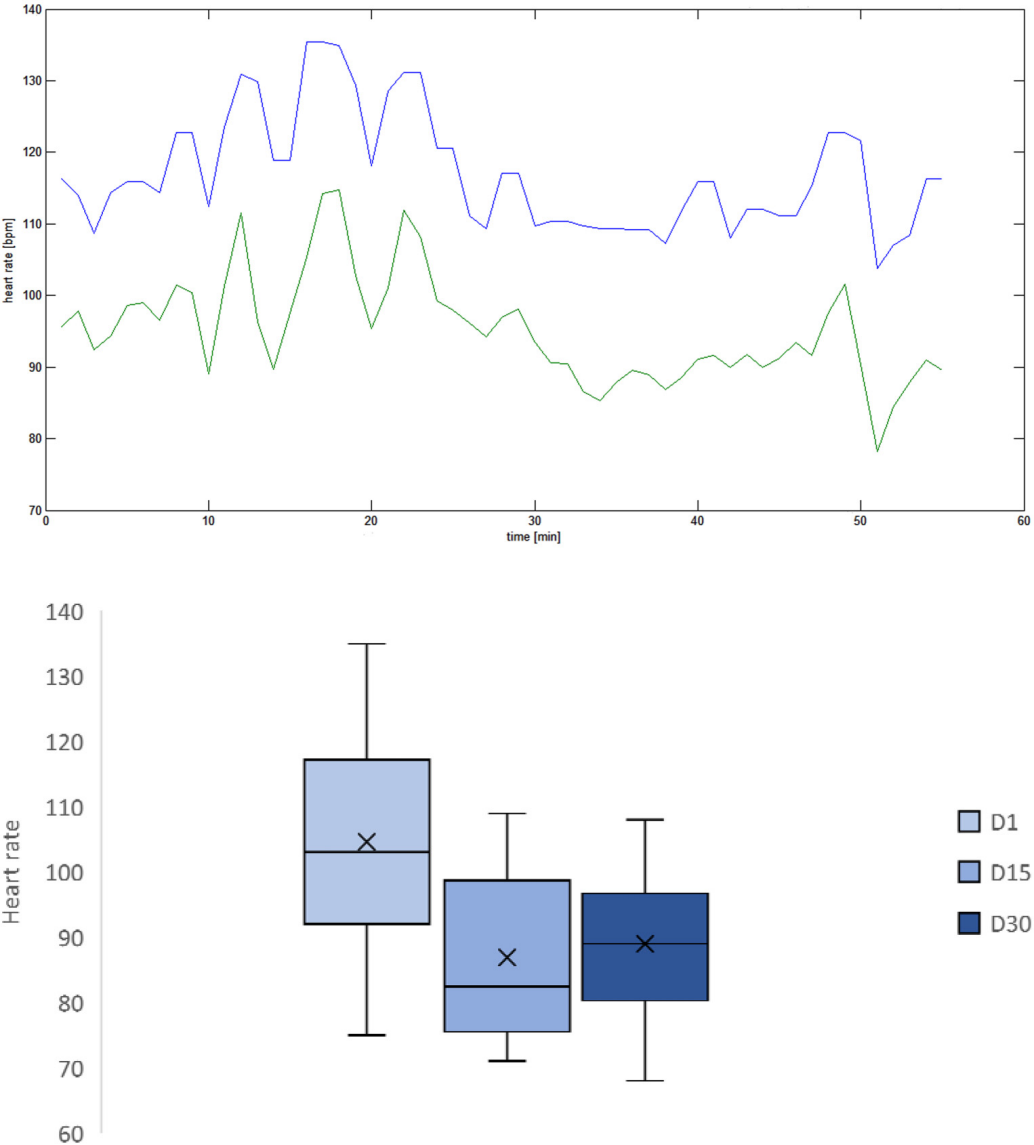
Example of heart rate variability in student 1 during microsurgical suture (upper picture) and average heart rate for students at day 1, day 15, and day 30 (lower picture). BPM: beats per minute.

The suture time (Figure 2) decreases significantly between days 1 and 15 (*p* = .002) but no longer between days 15 and 30 (*p* = .19). In comparison with the experts, the difference is highly significant at day 1 (*p* = .0006) and this difference remains significant at days 15 (*p* = .003) and 30 (.001). The heart rate (Figure 3) decreases significantly between days 1 and 15 (*p* = .01) but no longer between days 15 and 30 (*p* = .44).

**Figure 4.**
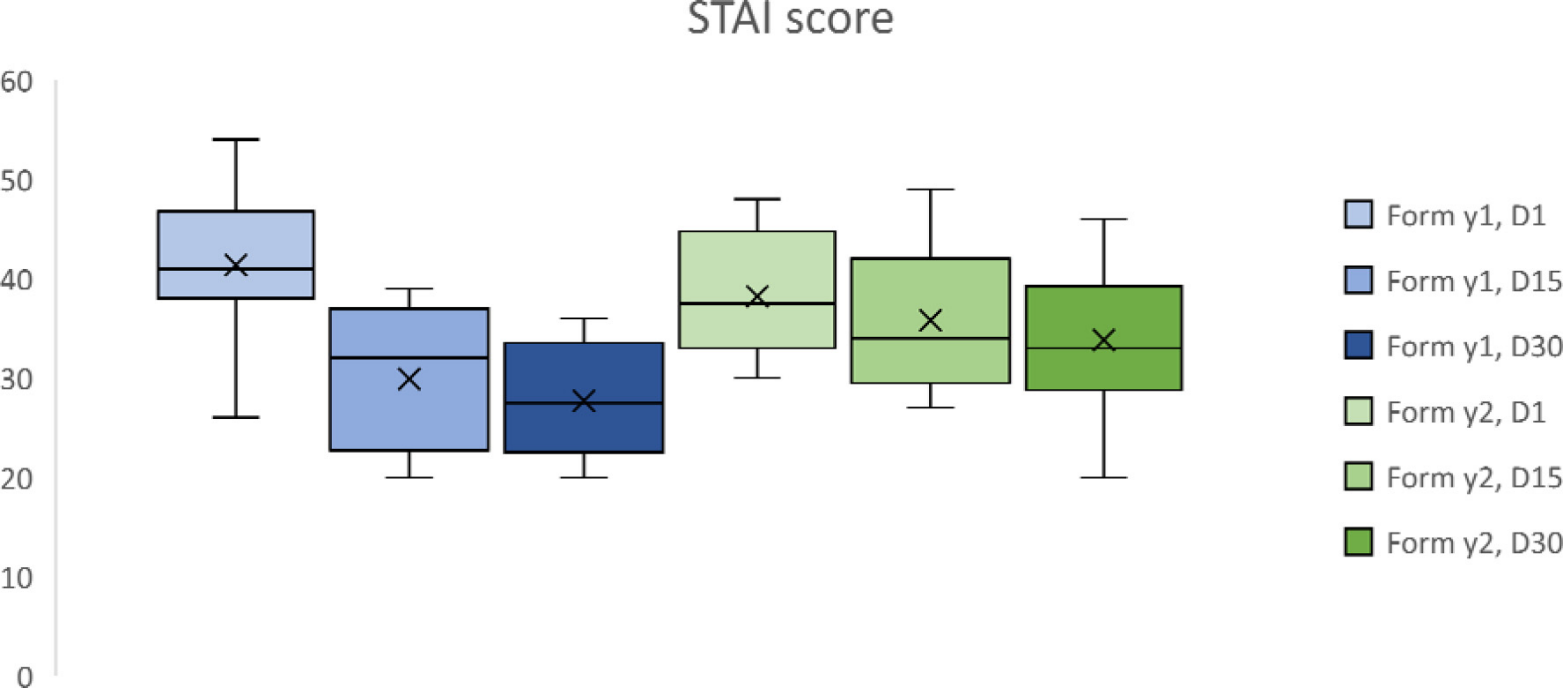
Results of state-trait anxiety inventory (STAI) score for students at day 1, day 15 and day 30. *p < .05.

The anxiety (Figure 4) specific to the task (STAI Form Y-1) decreases significantly between days 1 and 15 (p = 0.002) but no longer between days 15 and 30 (p = 0.23). General anxiety (STAI Form Y-2) decreases significantly between day 1 and day 15 (*p* = .03) and even more significantly between days 1 and 30 (*p* = .009).

**Figure 5.**
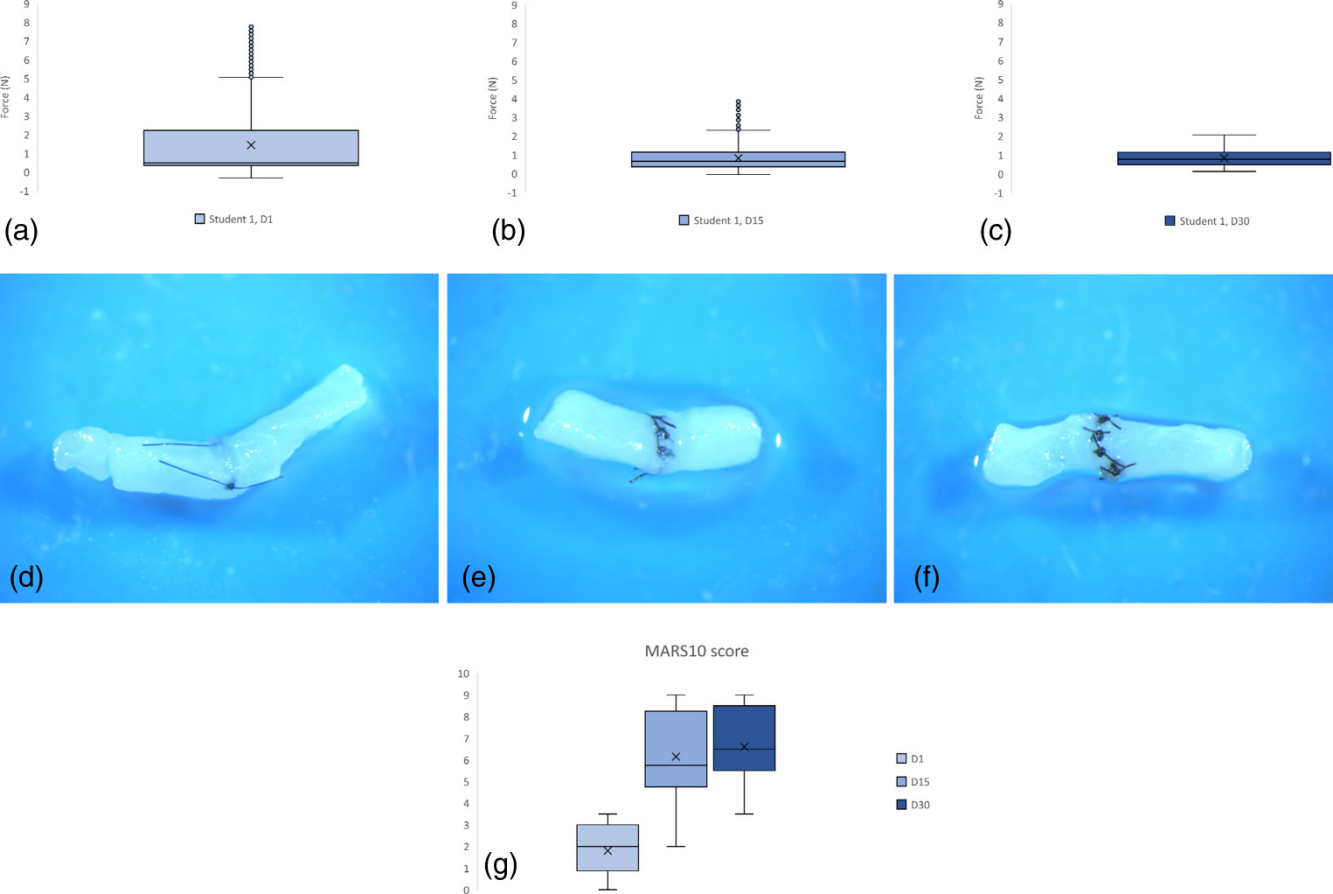
(a) Student 1, day 1, box plot of the force (N) applied (and recorded every 0.025 s) on the needle holder during knot tying on a chicken wing's artery. Box (IQR; 75th–25th percentile), central line (median; 50th percentile), lower whisker (fifth percentile), upper whisker (95th percentile) and *outliers (shown as blue circles)*. (b) Student 1, day 15 (c) Student 1, day 30 (d) result of suture for student 1 at day 1. MARS10 score = 3. (e) Result of the suture for student 1 at day 15. MARS10 score = 6 (f). Result of the suture for student 1 at day 30. MARS10 score = 8.5. (g) MARS10 score for all the students at day 1, day 15 and day 30 during the training period. *p < .05. MARS = Microsurgical Anastomosis Rating Scale; IQR = interquartile range.

The MARS10 score (Figure 5) has an excellent interobserver correlation (*r* = .95). The quality of the suture in the students increases significantly between days 1 and 15 (*p* = .006) but no longer between days 15 and 30 (*p* = .51).

## Discussion

We observed that the maximal, average, and standard deviation of the force applied to the needle holder significantly decreases between days 1 and 15 in microsurgery trainees. These parameters were significantly different between students and experts at day 1 only. Time to perform a simple microsuture on a chicken wing's artery decreases significantly between days 1 and 15 in students but the difference with experts is still significant at day 30. We conclude that the force parameters improve rapidly in students but that the suture time is probably the last factor to reach a steady state and requires more than a month of training.

Harada et al^23^ designed sensorized tweezers by attaching strain gauges to forceps’ tips and measured needle-gripping forces up to 5N. The force applied to the needle was assessed at different steps; during needle insertion, pushing, and extraction. They showed that skilled surgeons applied significantly less force during the needle extraction phase (*p* < .05). In the other phases, there was no statistically significant difference in the maximal gripping force between unskilled and skilled surgeons. Although not statistically significant, the force applied by skilled surgeons was somewhat lower in all phases. The findings in this study were not as significant as expected because an important part of their data could not be used as the artificial blood vessels were broken by the surgical instruments or because the suture was incomplete. In addition, they separated the task in steps that some surgeons did not carry out distinctly therefore, these data could not be analyzed.

A “smart” force-limiting instrument,^24^ which signals to the surgeon when the force applied on it exceeds a threshold of 0.3 N by producing vibrotactile feedback, was created from the modification of a blunt surgical dissector to allow its use in microneurosurgery. In laboratory and in vivo experiments,^25^ the maximal force exerted using a force-limiting modified dissector was significantly less than when using a standard dissector. The use of the force-limiting instrument did not significantly impede the surgical workflow. The development and use of force-sensing or -limiting instruments may be a useful tool for trainee surgeons and may improve patient safety in the clinical setting.

STAI is a commonly used scale for measuring state and trait anxiety.^21^ It has been widely adopted in clinical and research settings to diagnose anxiety and investigate its effects on performance. The scale consists of 2 questionnaires of twenty self-reported items each.

In our study, we observed that STAI Form-Y1 score decreased significantly between days 1 and 15. More surprisingly, the STAI Form-Y2 score, which assesses general anxiety in life, also decreased significantly between days 1 and 15. Since the students filled the forms immediately after completing the anastomosis, there might be an increase in self-confidence related to noticing some degree of progress.

Another study analyzed skills acquisition and stress adaptation in novice surgeons during laparoscopic surgery with an assessment after 2, 5, 8 h and then after 4 weeks free of training. It confirms that the STAI scores were significantly lower after training. The heart rate value and variability were lower after 5 and 8 hours of laparoscopic training compared to 2 hours.^26^ The STAI questionnaire is also a valid approach to evaluate stress during a procedure as the scores have been shown to be higher during a stressful surgery. There is a correlation between the STAI scores and objective measures (heart rate and salivary cortisol) for 70% of the procedures.^15^ These results suggest a correlation between psychological stress and the reaction of the autonomic nervous system. Our study also shows that heart rate decreases significantly during the learning process (Figure 3).

While there has been considerable research into motor learning in humans, the mechanisms behind movement acquisition and execution are still largely unknown. In many studies, motor skills acquisition was associated with a decrease in brain activity after training.^27^ This phenomenon is often described as “neural efficiency.”^28^

Popular evaluation tools of the quality of a surgical skill like the GRS (Global Rating Scale) or the University of Western Ontario Microsurgical Skills Acquisition/Assessment require an expert evaluating participant in real time, or a recorded microscope video feed of the participant's work.^5^ The MARS10 scale, which evaluates the result of the completed end-to-end arterial anastomosis (Stogowski et al),^22^ can be completed a posteriori. We observed an excellent interobserver correlation (*r* = .95) and the quality of the suture in the students increases significantly between day 1 and day 15 using MARS10.

## Conclusion

We studied subjective and objective parameters related to the relaxation state of students undergoing microsurgical training over a 4-week period. We observed a significant decrease in peripheral and central nervous system stress-related variables as the microsurgical skills improved. Relaxation parameters should be taken into consideration during microsurgical education and force-sensing microsurgical needle holder offers a new tool for the evaluation of relaxation and can function as a learning aid.
